# Attention-Deficit/Hyperactivity Disorder Medication Prescription Claims Among Privately Insured Women Aged 15–44 Years — United States, 2003–2015

**DOI:** 10.15585/mmwr.mm6702a3

**Published:** 2018-01-19

**Authors:** Kayla N. Anderson, Elizabeth C. Ailes, Melissa Danielson, Jennifer N. Lind, Sherry L. Farr, Cheryl S. Broussard, Sarah C. Tinker

**Affiliations:** ^1^Epidemic Intelligence Service, CDC; ^2^Division of Congenital and Developmental Disorders, National Center on Birth Defects and Developmental Disabilities, CDC; ^3^Division of Human Development and Disability, National Center on Birth Defects and Developmental Disabilities, CDC.

Attention-deficit/hyperactivity disorder (ADHD) is a neurodevelopmental disorder that affects individuals across the lifespan. ADHD medication use among pregnant women is increasing (*1*), but consensus about the safety of ADHD medication use during pregnancy is lacking. Given that nearly half of U.S. pregnancies are unintended (*2*), and early pregnancy is a critical period for fetal development, examining trends in ADHD medication prescriptions among reproductive-aged women is important to quantify the population at risk for potential exposure. CDC used the Truven Health MarketScan Commercial Database* for the period 2003–2015 to estimate the percentage of women aged 15–44 years with private employer-sponsored insurance who filled prescriptions for ADHD medications each year. The percentage of reproductive-aged women who filled at least one ADHD medication prescription increased 344% from 2003 (0.9% of women) to 2015 (4.0% of women). In 2015, the most frequently filled medications were mixed amphetamine salts, lisdexamfetamine, and methylphenidate. Prescribing ADHD medications to reproductive-aged women is increasingly common; additional research on ADHD medication safety during pregnancy is warranted to inform women and their health care providers about any potential risks associated with ADHD medication exposure before and during pregnancy.

CDC used the Truven Health MarketScan Commercial Database to examine outpatient pharmacy prescription drug claims for ADHD medications among reproductive-aged (15–44 years) women during 2003–2015. These data represent a convenience sample of persons with private employer-sponsored insurance and their dependents in the United States. Demographic data are available for all persons enrolled at any point during the year, regardless of whether a claim is filed, and are linkable to submitted outpatient pharmacy claims. This analysis was restricted to women aged 15–44 years with ≥11 months of enrollment in a private health insurance plan that included prescription drug coverage during the year of interest. Outpatient pharmacy claims for ADHD medications were identified using national drug codes, irrespective of the indication for use. Data were analyzed to assess the annual percentage of reproductive-aged women who filled any ADHD medication prescription during 2003–2015, as well as by age group, U.S. geographic region, and medication class. To examine time trends, the percentage change in the percentage of reproductive-aged women dispensed ADHD medications from 2003 to 2015 was estimated. Among women who filled at least one ADHD medication prescription in the given year, CDC examined the distribution of specific medications and average number of prescriptions filled per year.

Approximately 2.3–6.8 million privately insured reproductive-aged women constituted the analytic sample each year during 2003–2015 (median = 4.6 million). The percentage of reproductive-aged women with private employer-sponsored insurance who filled a prescription for any ADHD medication increased 344% from 2003 (0.9%) to 2015 (4.0%). The increase in the percentage of women prescribed ADHD medications was confined to a rise in the prescribing of stimulant medications^†^ (388% increase from 2003 to 2015); the percentage of women prescribed the nonstimulant medication atomoxetine was stable over time (0% change from 2003 to 2015) (Figure).

**FIGURE:**
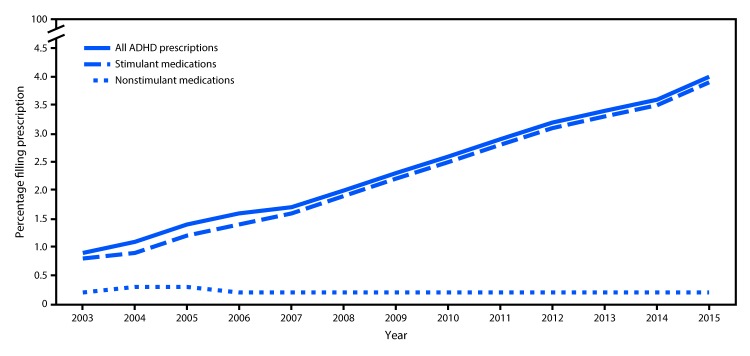
Percentage of women aged 15–44 years with private employer-sponsored insurance who filled one or more prescriptions for an attention-deficit/hyperactivity disorder (ADHD) medication, by medication class — United States, 2003–2015

The percentage of reproductive-aged women who filled a prescription for any ADHD medication increased over time for all age groups and geographic regions (Table 1). In 2015, the highest percentage of ADHD medication prescriptions filled among reproductive-aged women were for those aged 15–19 (5.4%), 20–24 (5.5%), and 25–29 (4.0%) years. From 2003 to 2015, the largest increase in ADHD prescriptions filled occurred among women aged 25–29 years (700%). In 2015, the highest percentage of ADHD medication prescriptions were filled by reproductive-aged women who resided in the South (4.8%) and North Central (4.0%) U.S. regions; the largest increase from 2003 to 2015 occurred in the South (380%).

**TABLE 1:** Percentage of women aged 15–44 years with private employer-sponsored insurance who filled a prescription for a medication commonly prescribed for attention-deficit/hyperactivity disorder (ADHD), by selected demographic characteristics — United States, 2003–2015

Characteristic	% by year													% Increase 2003 to 2015*
	2003	2004	2005	2006	2007	2008	2009	2010	2011	2012	2013	2014	2015	
**Age group (yrs)^†^**														
15–19	2.0	2.6	3.0	3.4	3.4	3.9	4.2	4.4	4.6	4.8	4.9	5.1	5.4	170
20–24	1.0	1.4	1.8	2.3	2.5	3.1	3.5	4.1	4.5	4.8	5.0	5.2	5.5	450
25–29	0.5	0.6	0.8	1.0	1.2	1.5	1.9	2.2	2.7	3.0	3.3	3.5	4.0	700
30–34	0.5	0.6	0.8	1.0	1.0	1.3	1.5	1.8	2.1	2.3	2.6	2.9	3.3	560
35–39	0.6	0.7	0.9	1.1	1.1	1.3	1.6	1.8	2.0	2.2	2.4	2.6	3.0	400
40–44	0.6	0.8	1.0	1.1	1.1	1.3	1.5	1.7	1.9	2.1	2.3	2.6	2.9	383
U.S. region^†,§,¶,^**														
Northeast	0.8	1.0	1.3	1.4	1.4	1.7	1.9	2.3	2.6	2.8	3.0	3.1	3.2	300
North Central	1.0	1.3	1.5	1.7	1.7	2.0	2.2	2.6	3.0	3.3	3.6	3.7	4.0	300
South	1.0	1.4	1.6	1.9	2.0	2.4	2.7	3.1	3.5	3.8	4.2	4.4	4.8	380
West	0.6	0.7	0.8	1.0	1.1	1.2	1.4	1.6	1.9	2.0	2.1	2.3	2.6	333
**Medication class**														
Any ADHD	0.9	1.1	1.4	1.6	1.7	2.0	2.3	2.6	2.9	3.2	3.4	3.6	4.0	344
Stimulant	0.8	0.9	1.2	1.4	1.6	1.9	2.2	2.5	2.8	3.1	3.3	3.5	3.9	388
Nonstimulant	0.2	0.3	0.3	0.2	0.2	0.2	0.2	0.2	0.2	0.2	0.2	0.2	0.2	0
**No. of eligible women^††^**	2,508,874	2,502,007	2,464,780	2,347,850	4,123,520	4,644,384	5,443,982	5,843,448	6,662,828	6,822,137	5,889,264	6,063,330	4,580,924	—

**Source:** Truven Health MarketScan Commercial Database.

* The same woman could be included in multiple years of data; the percentage change estimation describes the overall percentage change in the percentage of reproductive-aged women who filled ADHD medication prescriptions from 2003 to 2015 by each demographic characteristic.

^†^ Percentage with prescriptions dispensed was calculated among the total population of eligible women (i.e., women aged 15–44 years enrolled ≥11 member months per year in a plan that includes prescription drug coverage) who met the particular demographic characteristic for each age group and geographic region, respectively.

^§^ Among women eligible for the analytic sample, data for U.S. geographic region were missing for 0.2%–2.9%; data are not presented here.

^¶^ The U.S. region categories used by the Truven Health MarketScan Commercial Database align with the U.S. Census regions. The North Central region in the MarketScan Commercial Database is congruent with the Midwest Census region.

** *Northeast*: Connecticut, Maine, Massachusetts, New Hampshire, New Jersey, New York, Pennsylvania, Rhode Island, and Vermont; *Midwest*: Illinois, Indiana, Iowa, Kansas, Michigan, Minnesota, Missouri, Nebraska, North Dakota, Ohio, South Dakota, and Wisconsin; *South*: Alabama, Arkansas, Delaware, District of Columbia, Florida, Georgia, Kentucky, Louisiana, Maryland, Mississippi, North Carolina, Oklahoma, South Carolina, Tennessee, Texas, Virginia, and West Virginia; *West*: Alaska, Arizona, California, Colorado, Hawaii, Idaho, Montana, Nevada, New Mexico, Oregon, Utah, Washington, and Wyoming.

^††^ Women aged 15–44 years enrolled ≥11 member months per year in a plan that includes prescription drug coverage.

In 2015, among reproductive-aged women who filled any ADHD prescription, 60.8% filled a prescription for mixed amphetamine salts, 26.7% filled a prescription for lisdexamfetamine, and 18.1% filled a prescription for methylphenidate (Table 2). Among reproductive-aged women who filled any ADHD medication prescription in the given year, the percentage who filled a prescription for mixed amphetamine salts and lisdexamfetamine increased from 2003 to 2015, while the percentage who filled a prescription for methylphenidate and atomoxetine decreased over the same period. Among women who filled any ADHD medication prescription, the average number of prescriptions filled for any ADHD medication per year rose from an average of 5.5 prescriptions in 2003 (standard deviation [SD] = 4.4) to 7.2 in 2015 (SD = 5.1).

**TABLE 2:** Percentage of women who filled prescriptions for attention-deficit/hyperactivity disorder (ADHD) medications, by medication type, and average number of ADHD medication prescriptions filled per year, among women aged 15–44 years with private employer-sponsored insurance* who filled any ADHD prescription from outpatient pharmacies^†^ — United States, 2003–2015

ADHD medication^¶^	% by year^§^												
	2003	2004	2005	2006	2007	2008	2009	2010	2011	2012	2013	2014	2015
Amphetamine	0.0	0.0	0.0	0.0	0.0	0.0	0.0	0.0	0.0	0.0	0.0	0.0	0.3
Mixed amphetamine salts	44.6	45.4	49.7	54.6	57.0	56.1	55.8	56.5	57.3	58.0	59.4	60.3	60.8
Dexmethylphenidate	1.0	1.1	2.2	4.1	4.7	4.4	4.1	3.8	3.7	3.5	3.2	3.1	3.1
Dextroamphetamine	6.0	4.3	3.5	3.1	3.2	2.9	2.7	2.4	2.4	1.9	1.7	1.6	1.5
Lisdexamfetamine**	0.0	0.0	0.0	0.0	4.0	12.9	17.6	20.9	23.3	24.2	24.4	24.6	26.7
Methamphetamine	0.1	0.1	0.0	0.1	0.1	0.0	0.0	0.0	0.0	0.0	0.0	0.0	0.0
Methylphenidate	42.8	38.1	37.3	35.7	33.6	30.3	28.1	25.5	24.6	22.8	21.2	20.4	18.1
Pemoline**	1.1	0.7	0.4	0.1	0.0	0.0	0.0	0.0	0.0	0.0	0.0	0.0	0.0
Atomoxetine	20.6	24.5	19.7	13.7	10.9	9.2	7.5	6.5	5.5	4.9	4.4	4.1	3.8
**No. of eligible women with ≥1 ADHD prescription filled per year**	21,333	28,003	33,189	37,595	69,518	92,424	123,404	149,340	194,466	216,496	199,574	219,860	183,053
**Average no. of prescriptions filled per year (SD)^††^**	5.5 (4.4)	5. 5 (4.4)	5.6 (4.4)	5.9 (4.6)	6.0 (4.7)	6.1 (4.7)	6.3 (4.7)	6.4 (4.8)	6.5 (4.8)	6.7 (4.9)	6.9 (5.0)	7.1 (5.1)	7.2 (5.1)

**Source:** Truven Health MarketScan Commercial Database.

**Abbreviation:** SD = standard deviation.

* Women aged 15–44 years enrolled ≥11 member months per year in a plan that includes prescription drug coverage were defined as “eligible.”

^†^ The same woman could be included in multiple years of data.

^§^ Percentage of privately insured reproductive-aged women with each ADHD prescription medication dispensed was calculated among eligible women with at least one prescription filled for any ADHD medication in the given year.

^¶^ Not mutually exclusive; percentages might sum to >100% because multiple medications might have been prescribed to individual women within 1 calendar year. The first eight medications are stimulant ADHD medications and the last medication (atomoxetine) is a nonstimulant ADHD medication; these were the medications searched for in this analysis.

^**^Lisdexamfetamine was first approved by the FDA in 2007; pemoline was discontinued in 2005.

^††^ Among privately insured reproductive-aged women with at least one ADHD medication filled; this calculation is based on the average number of prescriptions filled each year from any type of ADHD medication.

## Discussion

The percentage of reproductive-aged women with private employer-sponsored insurance that included drug coverage who filled an ADHD medication prescription increased 344% from 2003 to 2015. In 2015, 4.0% of reproductive-aged women in this large convenience sample filled an ADHD medication prescription. A rise in stimulant ADHD medication prescriptions accounted for this increase; prescriptions for the nonstimulant atomoxetine have remained stable since 2003. The substantial increase in the percentage of reproductive-aged women filling ADHD medication prescriptions from 2003 to 2015, across age groups and U.S. geographic regions, is of public health concern given the high percentage of unintended pregnancies (*2*) and uncertainty concerning the safety of ADHD medication exposure before and during pregnancy (*3*). In studies with samples of U.S. pregnant women, ADHD medication use estimates have ranged from 0.4% (2000–2013 data) (*4*) to 1.3% (2013 data) (*1*). Although evidence is limited and findings are mixed (*3*), ADHD medication use during pregnancy might be linked to increased risk for poor pregnancy outcomes, including spontaneous abortion (*5*,*6*). The safety of ADHD medications with regard to risk for birth defects is largely unknown, with only one sufficiently powered published study (*4*).

ADHD medication prescription trends among reproductive-aged women in non-U.S. populations align with CDC’s findings that an increased percentage of women are filling ADHD medication prescriptions, with the highest percentage among younger reproductive-aged women. In an analysis of 2003–2008 data from the United Kingdom (*7*), the prevalence of ADHD medication prescriptions increased over time among women aged 18–24 years (from 0.12 to 0.34 per 1,000 women) and women aged 25–45 years (from 0.01 to 0.05 per 1,000 women). In an analysis of Canadian adults during 2005–2015 (*8*), the prevalence of ADHD medication prescriptions increased over time for men and women aged 18–25 years (from 0.7% in 2005 to 3.2% in 2015) and 26–35 years (from 0.3% in 2005 to 1.6% in 2015). CDC’s estimates were higher than those from the United Kingdom and Canadian data sets, which might reflect higher ADHD medication prescribing in the United States or differences in the types of ADHD medications either prescribed across countries or included in the analyses. Most adult ADHD medication use prevalence estimates use older data (*5*,*7*), whereas results from this analysis demonstrate a continued increase in ADHD medication prescribing into 2015.

CDC’s findings indicate that mixed amphetamine salts, lisdexamfetamine, and methylphenidate are among the ADHD medications most commonly prescribed to privately insured U.S. reproductive-aged women. In the United States, mixed amphetamine salts and methylphenidate are the most frequently prescribed ADHD medications among children (*9*) and pregnant women (*1*). Data from this analysis similarly suggests that mixed amphetamine salts and methylphenidate are two of the three most commonly prescribed medications among reproductive-aged women. However, in this analysis, lisdexamfetamine, which was approved by the Food and Drug Administration in 2007, was the second most commonly prescribed medication among reproductive-aged women. This is noteworthy given that most analyses that have examined ADHD medication safety among women before and during pregnancy have not included lisdexamfetamine as a medication of interest (*3*–*6*).

The findings in this report are subject to at least four limitations. First, although this analysis included 2.3–6.8 million reproductive-aged women per year, data are from a convenience sample of privately insured women with prescription drug coverage. Approximately 45% of U.S. births occur to women with Medicaid coverage (*10*); ADHD medication prevalence estimates might differ between publicly and privately insured women of reproductive age. Second, data are based on outpatient pharmacy claims and no information is available on women who paid for prescriptions out-of-pocket or who obtained ADHD medications from someone other than their prescribing physician. Third, although data represent ADHD medications dispensed, verification that women took the medications after the prescription was filled is not available. Finally, this analysis focused on women aged 15–44 years and did not identify pregnant women or women’s risk for pregnancy.

This analysis used a large database to estimate the percentage of privately insured reproductive-aged women who filled an ADHD medication prescription during 2003–2015. The increasing trend toward prescribing ADHD medications to reproductive-aged women highlights the importance of research examining ADHD medication safety in this population, including safety before and during pregnancy. CDC’s Treating for Two: Safer Medication Use in Pregnancy initiative (https://www.cdc.gov/treatingfortwo) helps address this need by conducting research on medication safety before and during pregnancy to help women and their health care providers make evidence-based decisions regarding the risks and benefits of pharmacologic and behavioral treatment options for common conditions, including ADHD.

**Summary**

**What is already known about this topic?**

Attention-deficit/hyperactivity disorder (ADHD) medication use has increased among U.S. pregnant women, and consensus about its safety during pregnancy is lacking. Given that half of U.S. pregnancies are unintended, ADHD medication use among reproductive-aged women might result in early pregnancy exposure, a critical period for fetal development.

**What is added by this report?**

The percentage of privately insured reproductive-aged women who filled a prescription for an ADHD medication increased 344% from 2003 (0.9%) to 2015 (4.0%). ADHD medication prescriptions increased across all age groups and U.S. geographic regions, and the increase was confined to stimulant medications.

**What are the implications for public health practice?**

ADHD medication prescriptions are increasingly common among privately insured, reproductive-aged women. Additional research on ADHD medication safety among this population, including safety before and during pregnancy, could help women and their health care providers make evidence-based decisions concerning the risks and benefits of pharmacologic and behavioral treatment options for common conditions, including ADHD.

## Footnotes

* Truven Health Analytics, Atlanta, Georgia.

† In this analysis, stimulant medications include amphetamine, mixed amphetamine salts, dexmethylphenidate, dextroamphetamine, lisdexamfetamine, methamphetamine, methylphenidate, and pemoline.
